# Epidermoid cyst of the floor of the mouth: two case reports

**DOI:** 10.1186/1757-1626-2-9360

**Published:** 2009-12-20

**Authors:** Paraskevi Tsirevelou, Mattheos Papamanthos, Paschalis Chlopsidis, Ifigenia Zourou, Charalampos Skoulakis

**Affiliations:** 1 ENT, "Achillopouleion" General Hospital of Volos, Polymeri 134, 38222 Volos, Greece; 2 Dental Care "Achillopouleion" General Hospital of Volos, Polymeri 134, 38222 Volos, Greece; 3 Pathology Department, "Achillopouleion" General Hospital of Volos, Polymeri 134, 38222 Volos, Greece

## Abstract

**Introduction:**

Epidermoid cysts that appear in the midline floor of the mouth are, usually, a result of entrapped ectodermal tissue of the first and second branchial arches, which fuse during the third and fourth weeks in utero. The incidence in the floor of the mouth of the oral cavity is rare and development sites are the sublingual, submaxillary and submandibular spaces.

It was present two cases of epidermoid cyst of the floor of the mouth and discussed the different surgical approaches for this lesion.

**Cases presentation:**

Two cases of midline epidermoid cysts of the floor of the mouth are presented, evaluating the different surgical approaches. The preoperative assessment was made using ultrasonography and computed tomography in both cases. Regarding surgical techniques used, a transcutaneous approach was adopted when the cysts were under the geniohyoid muscle and a midline incision of the oral mucosa along the lingual frenulum was used for sublingual cysts. During the postoperative course, there were no complications, except for mild edema in one case. Follow-up ranged between 5 months and 4 years; no recurrence or malignant changes were observed.

**Conclusions:**

Surgery of epidermoid cyst of the floor of the mouth is the treatment of choice. Access depends on the lesion's location in relation to the mylohyoid or geniohyoid muscles. If the cyst is located over the mylohyoid, surgery is carried out only through the oral cavity, whereas the extraoral incision was necessary only when the cysts were under the geniohyoid muscle.

## Introduction

Dermoid and epidermoid cysts are uncommon developmental cystic malformations termed dysontogenetic cyst. Most clinicians and researchers believe that dermoid and epidermoid cysts that appear in the midline floor of the mouth are a result of entrapped ectodermal tissue of the first and second branchial arches, which fuse during the third and fourth weeks in utero. A second theory suggests that midline dermoid and epidermoid cysts may be a variant of the thyroglossal duct cyst with ectodermal elements predominating [1].

Epidermoid and dermoid cysts constitute 1.6 to 6.9% of all cysts in the head and neck area [2]. Common location sites are the orbit, calvarial diploic space and intracranially. The incidence in the floor of the mouth of the oral cavity is rare and represented less than 0,01% of all cysts of the oral cavity. Sublingual, submaxillary and submandibular spaces are common localization in the floor of the mouth [3].

Epidermoid cysts generally present slow and progressive growth, and even if they are congenital, the diagnosis is commonly possible in the second or third decade of life [4]. They appear as painless, asymptomatic mass, slowly increasing in size, usually located in the midline, above or below the mylohyoid muscle. Treatment of epidermoid cysts of the floor of the mouth is surgical and can be intraoral or extraoral according to the localization and the size of the lesion.

In the current report we describe two cases of epidermoid cyst of the floor of the mouth, and discussed the differences in surgical approaches.

## Case presentation

### Case report 1

**Figure 1:**
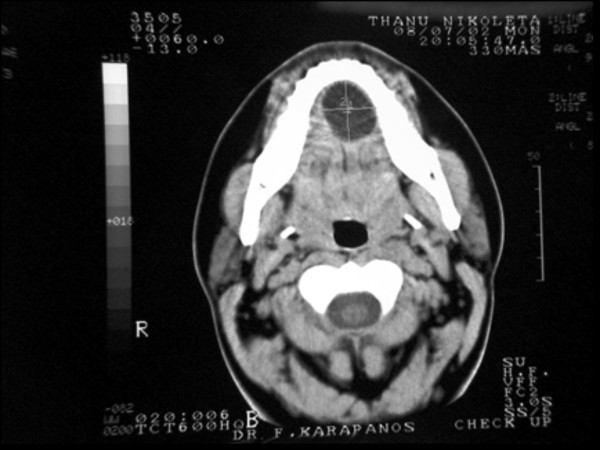
**Axial CT section of the floor of the mouth showed a 1.38 × 1.18 in hypodense, non-enhancing mass located within the sublingual space, over the geniohyoid muscle**.

**Figure 2:**
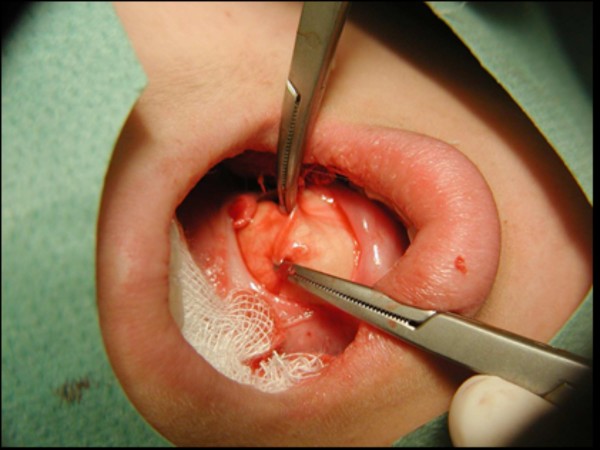
**The elliptical incision was made on the floor of the mouth**.

**Figure 3:**
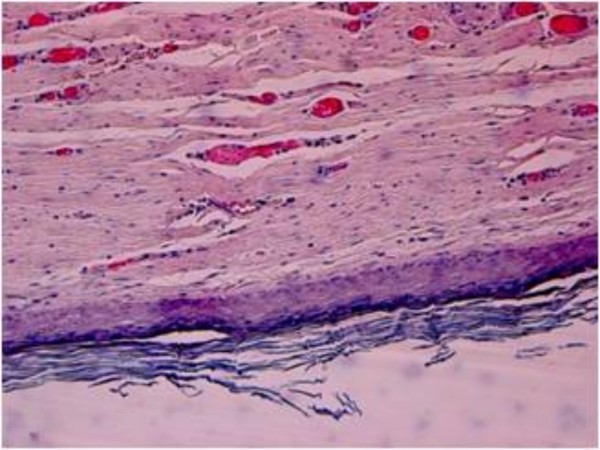
**Histopathological examination of the surgical specimen revealed cystic cavity lining by keratinized stratified squamous epithelium and lumen containing keratin (Haematoxilin and Eosin, magnification × 100)**.

A 14-year-old girl from island of Crete, in Greece, presented a soft, painless, movable and touchable intraoral swelling, which was initially small and increased in size over duration of 10 months. On examination of the oral cavity, there was a swelling in the left side of the floor of the mouth, pushing the tongue to the right side. Upon bimanual palpation a dough like, non-tender mass was felt. There was no evidence of cervical lymphadenopathy. The skin and mucosa over the swelling were intact and normal. A contrast enhanced CT scan showed a 1.38 × 1.18 in, hypodense, non-enhancing mass arising within the sublingual space (Figure 1). The mass extended into the floor of the mouth. Due to the period of evolution (only 10 months) and the absence of pain and of infectious foci in the oral cavity, the hypothesis of an infection was discarded. The hypothesis of a malignancy was also discarded due to the clinical aspect of the lesion and the absense of lymphadenopathy. So with the tentative diagnosis of ranula, aspiratory punction was carried out. This revealed the presence of epithelial remnants, desquamated tissue and cellular debris which pointed to a new diagnostic hypothesis of epidermoid cyst. The cyst was completely removed by surgical excision. The elliptical incision was made on the floor of the mouth, followed by blunt dissection (Figure 2). The lesion was found sitting on top of the genioglossus muscle. Histopathological examination revealed a cyst lined by keratinized stratified squamous epithelium (Figure 3) The diagnosis of epidermoid cyst was made. The postoperative course in healing was free of complications. The patient was followed up for two years with clinical examination and ultrasonography twice a year with no signs of recurrence.

### Case report 2

**Figure 4:**
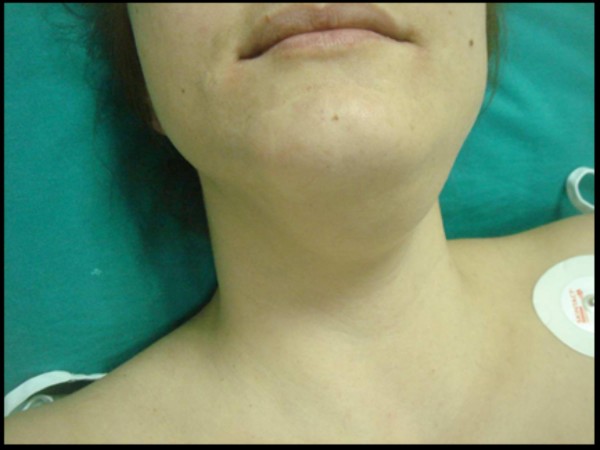
**The 35 year-old female presented with a submandibular left-sided neck swelling**. The skin over the swelling was intact and normal.

**Figure 5:**
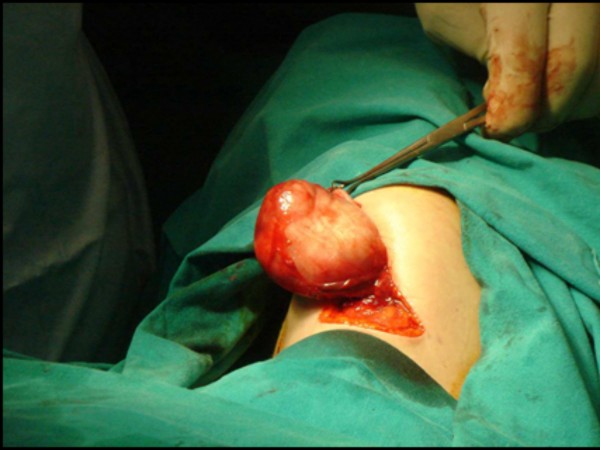
**The lesion was removed by external approach through an transverse incision in the left submandibular area extending beyond the midline to the opposite side**.

**Figure 6:**
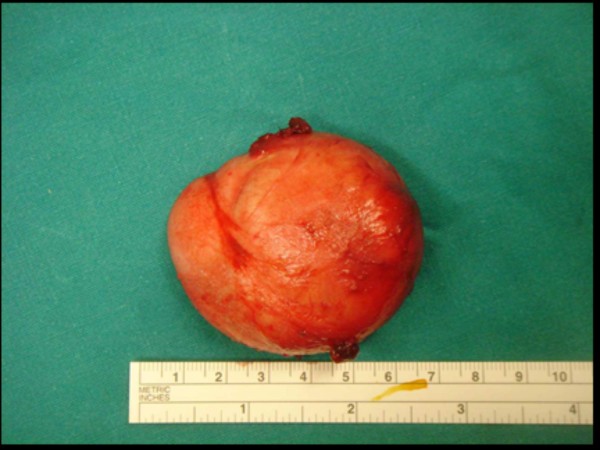
**The 2.17 in intact epidermoid cyst of case 2 after surgical removal**.

A 35 year-old female patient from the greek city of Volos presented to us with a left-sided neck swelling (Figure 4) and an intraoral swelling which was initially small and increased in size over a duration of 6 months. The patient also complained of dysphagia, dysarthria and dyspnoea on exertion. On examination of the neck there was a well circumscribed swelling in the left submandibular region. On examination of the oral cavity, there was a swelling in the left side of the floor of the mouth and in submandibular region. Upon bimanual palpation a dough like, non-tender mass could be felt. The skin and mucosa over the swelling were intact and normal. A contrast enhanced CT scan showed a large, hypodense, non-enhancing mass arising within the submandibular space. Our first hypothesis was that of ranula, since the clinical aspect was compatible with ranula and because ranulas are far more common than epidermoid cysts. The hypothesis of an infection was discarded due to the period of evolution (6 months) and the absence of pain and of intraoral infectious foci. Malignant tumor was ruled out in view of the lesion's clinical aspect and the absence of lymphadenopathy, although the latter is admittedly an imprecise indicator of malignancy. Fine-needle aspiration biopsy was nondiagnostic. Under general anaesthesia, a transverse incision was made in the left submandibular area extending beyond the midline to the opposite side (Figure 5). This was carried through skin, subcutaneous tissue and platysma. Blunt dissection was utilized to free the mass, which was removed intact after which the right submandibular gland was reposited within its capsular bed (Figure 6). The wound was sutured in layers and a corrugated rubber drain was placed in position. Histopathological examination revealed the same features of the case 1. Also, the histological diagnosis was the epidermoid cyst. Re-examination of the patient 1 week and 4 weeks postoperatively found patient without swelling and free of complaints. A follow up of three years with clinical examination and ultrasonography twice a year was performed and it found the patient with no signs of recurrence.

## Discussion

Epidermoid cysts of the floor of the mouth are considered rare. New and Erich (1937) reported 24 (1.6%) epidermoid cysts occuring at the floor of the mouth out of 1495 cases of dermoid cysts seen at the Mayo Clinic.

In 1955, Meyer updated the concept of dermoid cyst to describe three histological variants: the true dermoid cyst, the epidermoid cyst and the teratoid variant. True dermoid cysts are cavities lined with epithelium showing keratinization and with identifiable skin appendages such as pilous follicles, and sudoriparous and sebaceous glands on the cyst wall. Epidermoid cysts are lined with simple squamous epithelium with afibrous wall and no attached structures. The lining of teratoid cysts varies from simple squamous to a ciliate respiratory epithelium containing derivates of ectoderm, mesoderm and/or endoderm. All three histological types contain a thick, greasy-looking material [5].

Most patients with epidermoid cyst are in the range between 10 and 35 years of age [6]. In a series of 16 cases, the mean age is 27.8 years and the ratio of men/women is 3:13, although previous papers have found no difference by gender while others have found predominance of women. Growth of the cyst may be constrained by hormonal stimulus during puberty, producing a hypersecretion of fat, which would explain the greater incidence in the young adult stage (16-40 years of age) [5].

Anatomic classification divides the epidermoid cysts of the floor of the mouth into three groups according their relation to the muscles of the floor of the mouth: sublingual or median genioglossal cysts, located above the geniohyoid muscles; median geniohyoid cysts, located in the submental region between the geniohyoid and the mylohyoid muscles; and lateral cysts, located in the submaxillary region [4]. The cystic mass can vary in size from a few millimeters up to 4.72 in in diameter [7].

The size and the location of the epidermoid cyst are the cause of the clinical manifestations. Cystic lesions developing above the mylohyoid muscle have the potential to displace the tongue toward the palate and subsequently create difficulty with mastication, speech, and possibly breathing. Cysts developing below the mylohyoid often produce a submental or submandibular swelling [7]. In our first case the sublingual swelling suggests that the lesion was above the mylohyoid muscle, which is the most common location. The mass was located in the left side of the floor of the mouth, pushing the tongue to the right side. The second patient had a well circumscribed swelling in the left submandibular region. It was complaining of dysphagia, dysarthria and dyspnoea on exertion.

When dealing with swellings in the floor of the mouth and neck region, 4 main groups of lesions should be considered: infections, tumors, mucous extravasation phenomena and anatomic abnormalities arising during embryonic development. In our cases, the hypothesis of an infection was discarded due to the period of evolution and the absence of pain and of intraoral infectious foci. Malignant tumor was ruled out in view of the lesion's clinical aspect and the absence of lymphadenopathy, although the latter is admittedly an imprecise indicator of malignancy. It was possible then left with two main diagnostic possibilities: a mucous extravasation phenomenon and an anatomic abnormality. Because the clinical aspect was compatible with ranula and because ranulas are far more common than epidermoid cysts, this was our first hypothesis. In some instances, where the differential diagnosis of sublingual swellings is more challenging, imaging techniques may be used for preoperative diagnosis and surgical planning. Fine-needle aspiration is not always diagnostic. Magnetic resonance imaging (MRI) and computed tomography (CT) allow more precise localization of the lesion, and also enable the surgeon to choose the most appropriate approach. Thus, microscopic examination will always be required following excision of the lesion [8].

Surgical enucleation is the only effective treatment for these kinds of lesions. Several techniques are reported in the literature, which may be divided into intraoral and extraoral techniques depending on which approach is used.

In the case of an intraoral approach, a midline vertical, mucosal incision is performed along the ventral surface of the tongue; however, only small cysts can be enucleated using this kind of incision [9]. Lowry et al. [10] describe a bilateral incision along the mandibular ridge crest, Brusati et al. [11] propose a midline glossotomy, and Di Fransesco et al [12] describe a modification of this surgical technique consisting of an extension of this incision along the ventral surface of the tongue associated with partial evacuation of the epidermoid cyst. The latter two techniques, according to Longo et al [4] allow to obtain a very good approach to the cyst and to obtain adequate surgical control of the lesion in the event of median cysts located above the geniohyoid muscles.

The transcutaneous approach consists of a submental incision and a sharp, blunt dissection to reach and enucleate the lesion [10]. Mc Gregor [13] describes a symphyseal mandibular osteotomy to enucleate a very large sublingual dermoid cyst. The extraoral approach is generally preferred in the case of median geniohyoid or very large sublingual cysts, whereas the intraoral approach is typically used for smaller sublingual cysts.

In the current first case, excision was achieved without major complications by employing intraoral access under general anestesia. The elliptical incision was made on the floor of the mouth, followed by blunt dissection. The lesion was found sitting on top of the genioglossus muscle. In the current second case we adopted a transcutaneous approach. Under general anaesthesia, a transverse incision was made in the left submandibular area extending beyond the midline to the opposite side. This was carried through skin, subcutaneous tissue and platysma. Blunt dissection was utilized to free the mass, which was removed intact after which the right submandibular gland was reposited within its capsular bed. The wound was sutured in layers and a corrugated rubber drain was placed in position. The postoperative course does not present any kind of problem because there is little alteration in function, edema is generally modest, and complications are unusuall.

Prognosis is very good, with a very low incidence of relapse, usually related to bone remnant to the genial tubercles or to the hyoid bone. Malignant changes have been recorded in dermoid cysts by New and Erich [14] but not in the floor of the mouth, although a 5% rate of malignant transformation of oral dermoid cysts has been reported by other authors, but only for the teratoid type [15].

## Conclusions

In conclusion, it was described two cases of epidermoid cysts successfully diagnosed and managed by following simple yet effective steps. The appropriate imaging techniques are very effective in preoperative. Fine needle aspiration cytology of the mass is not always diagnostic. Differential diagnosis includes infections, tumors, mucous extravasation phenomena and embryonic abnormalities. Surgical excision is the treatment of choice. In cases of sublingual cysts, in our experience, the intraoral approach is effective in the treatment of large lesions, as it leads to very good cosmetic and functional results. The extraoral incision is mandatory only when the cyst lies under the geniohyoid muscle.

## Consent

Written informed consent was obtained from the patient for publication of this case report and any accompanying images. A copy of the written consent is available for review by the Editor-in-Chief of this journal.

## Competing interests

The authors declare that they have no competing interests.

## Authors' contributions

MP conceived of the study, and participated in its design and coordination and helped to draft the manuscript. PT carried out the drafting of the manuscript and contributed in acquisition of data. PC has made substantial contributions to collection, acquisition and interpretation of data. IZ performed the histopathological examination. CS had the general supervision and have given final approval of the version to be published. All authors read and approved the final manuscript.
